# Are Ants Civilized?

**Published:** 1878-01

**Authors:** 


### Are Ants Civilized?

The October number of the "Quarterly Journal of Science" contains an article on "Our Six-footed Rivals," the ants, which may well cause us to believe that we are not the only rational and civilized beings on this globe.

Let us suppose that we were suddenly informed, on good authority that there existed a race of beings who lived in domed habitations, aggregated together so as to form vast and populous cities, that they exercised jurisdiction over the adjoining territory, laid out regular roads, executed tunnels underneath the beds of rivers, stationed guards at the entrance of their towns, carefully removed any offensive matter, maintained a rural police, organized extensive hunting expeditions, at times even waged war upon neighboring communities, took prisoners and reduced them to a state of slavery; that they not merely stored up provisions with due care, but that they kept cattle and even cultivated the soil and gathered in the harvest. We should unquestionably regard these creatures as human beings who had made no small progress in civilization, and should ascribe their actions to reason.

Among the *hymenoptera* the lead is undoubtedly taken by the ants, which, like man, have a brain much more highly developed than that of the neighboring inferior groups. Perhaps the most elevated of the formicide family is the agricultural ant of western Texas. This species is, save man, the only creature which does not depend for its sustenance on the products of the chase or the spontaneous fruits of the earth. A colony of these ants will clear a tract of ground, some four feet in width, around their city, and remove all plants, stone, and rubbish. A species of minute grain, resembling rice, is sown therein and the field is carefully tended, kept free from weeds, and guarded against marauding insects. When mature, the crop is reaped and the seeds dried aud carried into the nest. If this is done near a larger city the latter regard it as an intrusion, and a fierce warfare results, which ends in the total destruction of one or the other side. The queens are treated with great attention and installed in royal apartments.

The ant government is communistic. In a formicary there is no trace of private property; the territory, the buildings, the stores, the booty, exist equally for the benefit of all. The family among them scarcely exists. Rarely is the union of the male and female extended beyond the actual intercourse, all provision for the future young devolving upon the latter alone, the former being speedily killed, as he is no longer of any use. The females are larger, stronger, and more long-lived. The workers and fighters are sexless; to them belongs the real government of the ant-hill, and they provide for its enlargement, well being, and defence.

Ants are sometimes very stupid in regard to small things, but in many instances they display remarkable sagacity. Mr. Belt, in his "Naturalist in Nicaragua," tells of a column of ants who were crossing a watercourse by a small branch not thicker than a goose quill. They widened this natural bridge to three times its width by a number of ants clinging to it and to each other on each side, over which the column passed four deep, thus effecting a great saving of time. Again, the *eciton legionis*, when attacking the hill of another species, digs mines and passes the pellets of earth from ant to ant until placed at a sufficient distance outside to prevent it rolling back into the hole.—*Medical Bi-Weekly.*

